# *Notes from the Field:* Detection of Medetomidine Among Patients Evaluated in Emergency Departments for Suspected Opioid Overdoses — Missouri, Colorado, and Pennsylvania, September 2020–December 2023

**DOI:** 10.15585/mmwr.mm7330a3

**Published:** 2024-08-01

**Authors:** Evan S. Schwarz, Jennie Buchanan, Kim Aldy, Joshua Shulman, Alex Krotulski, Sara Walton, Barry Logan, Paul Wax, Sharan Campleman, Jeffrey Brent, Rachel Culbreth, Alex F. Manini, Toxicology Investigators Consortium Fentalog Study Group

**Affiliations:** ^1^University of California, Los Angeles, Los Angeles, California; ^2^Denver Health and Hospital Authority, Denver, Colorado; ^3^American College of Medical Toxicology, Phoenix, Arizona; ^4^Baylor University Medical Center, Dallas, Texas; ^5^University of Pittsburgh School of Medicine, Pittsburgh, Pennsylvania; ^6^Center for Forensic Science Research and Education, Fredric Rieders Family Foundation, Horsham, Pennsylvania; ^7^NMS Labs, Horsham, Pennsylvania; ^8^University of Colorado School of Medicine, Aurora, Colorado; ^9^Department of Emergency Medicine, Icahn School of Medicine at Mount Sinai, NYC Health + Hospitals, Elmhurst, New York; ^10^Mount Sinai Center for Research on Emerging Substances, Poisoning, Overdose, and New Discoveries (RESPOND), New York, New York.

**Summary**

**What is already known about this topic?**

Medetomidine, a veterinary anesthetic drug, is an emerging adulterant detected in illicit drugs, drug paraphernalia, and overdoses.

**What is added by this report?**

During August 2022–July 2023, medetomidine was detected among five patients along with xylazine, fentanyl, and illicit opioids. No permanent sequelae were reported. All patients received naloxone; however, only two received naloxone kits at discharge, and only one was referred for addiction treatment.

**What are the implications for public health practice?**

Medetomidine is an emerging adulterant detected in illicit drugs; further investigation is important to better understand the clinical effects of medetomidine and other novel adulterants. Programs to improve addiction treatment and naloxone distribution are needed.

Medetomidine, a canine veterinary agent used for its anesthetic and analgesic properties, is an emerging adulterant detected in illicit drugs and drug paraphernalia. Medetomidine is a racemic mixture of two optical isomers, levomedetomidine and dexmedetomidine; the pharmacologic effects are caused by dexmedetomidine, an alpha-2 agonist similar to xylazine (*1*). Medetomidine is not approved for human use. Xylazine, a well-described opioid adulterant, is associated with multiple adverse effects, including soft tissue wounds* (*2*). Whereas xylazine is not approved for human use, dexmedetomidine is used in hospitals as a sedative and analgesic. Dexmedetomidine is considered safe for sedation during certain procedures^†^ (*3*) and is not associated with wounds when administered intravascularly or intramuscularly in hospitals. Medetomidine has been detected in samples of street-level drugs beginning in 2022 (*4*). Medetomidine is increasingly found in the drug supply across the United States and Canada and was associated with overdoses in April and May 2024 in Philadelphia, Pittsburgh, and Chicago.^§^ The recent overdose outbreaks in Philadelphia, Pittsburgh, and Chicago highlight the need to understand symptoms associated with medetomidine use and overdose. This report describes the detection of medetomidine from illicit agents among patients evaluated in an emergency department (ED) after suspected opioid overdoses, none of whom had received medetomidine as part of clinical care.

## Investigation and Outcomes

Data for this analysis are from the Toxicology Investigators Consortium Fentalog Study Group, a 2020–2025 study of patients aged >18 years evaluated in 10 EDs in nine states after a suspected opioid overdose as part of ongoing activities to determine the role and prevalence of novel substances in these overdoses. Comprehensive toxicologic testing for the presence of approximately 1,200 drugs and metabolites, including an array of novel psychoactive substances and adulterants, was performed on residual blood samples using liquid chromatography quadrupole time-of-flight mass spectrometry. Patients who received a positive medetomidine test result were included. Additional case information was obtained through chart review.^¶^ A waiver of consent was obtained for specimen collection. This activity was approved centrally by Western Institutional Review Board, and locally at each participating institution.

During September 2020–December 2023, a total of 1,331 blood samples** collected from persons evaluated in participating EDs for a suspected opioid overdose were analyzed. Medetomidine was detected among five patients (0.4%) from three states (Missouri, Colorado, and Pennsylvania) during August 2022–July 2023 (Table). Patients A and B were initially hypotensive (systolic/diastolic blood pressures = 64/37 mmHg and 96/60 mmHg, respectively), but neither had bradycardia. Four patients were intentionally using opioids recreationally. All patients received naloxone (median total dose = 2 mg), one of whom received an infusion. All patients’ neurologic exams were normal by 4 hours after arrival in an ED. Patient C, a man, aged 30–39 years, had an elevated troponin concentration but was discharged 4 hours after arrival. Patients A and B initially had acidosis (pH = 7.29 and 7.19, respectively). Patient B, a woman, aged 20–29 years, experienced respiratory and hemodynamic complications shortly after arrival and received positive test results for methamphetamine and olanzapine, but negative test results for opioids; she was hospitalized for 18 days. Comprehensive blood testing confirmed the presence of fentanyl among three patients, illicit benzodiazepines among three, stimulants among three, xylazine among three, and nitazene opioids among two. Only two patients were documented to have received naloxone kits at discharge, and only one was referred for addiction treatment.

**TABLE:** Characteristics of patients with detection of medetomidine during emergency department visits after suspected opioid overdoses — Missouri, Colorado, and Pennsylvania, September 2020–December 2023

Characteristic	Patient				
	A	B	C	D	E
Age group, yrs; sex	40–49; man	20–29; woman	30–39; man	20–29; man	20–29; man
Date of detection	Mar 2, 2023	Jul 12, 2023	Apr 5, 2023	Aug 8, 2022	Aug 9, 2022
History of illicit opioid use in the previous 30 days	Yes	Unknown	Yes	Yes	Yes
History of other substance use	Alcohol and tobacco	Sedatives and stimulants	Unknown	Ethanol	Ethanol, cannabis, and stimulants
Psychiatric history	Anxiety and depression	Unknown	None	Schizophrenia	Bipolar disorder
No. of naloxone doses received	5*	1	1	1	3
Received initial naloxone dose before ED arrival	Yes	Yes	Yes	No	Yes
Total naloxone dosage received	3.95 mg*	2 mg	2 mg	0.4 mg	6 mg
Received prehospital CPR	Yes	No	No	No	No
Precipitated withdrawal^†^ after receiving naloxone	No	No	No	Yes	No
Highest level of hospital care	ICU	ICU	ED	ED	ED
Disposition	Self-discharged (left AMA)	Transferred to psychiatric facility	Discharged	Self-discharged (left AMA)	Discharged
Length of stay, hrs	19	427 (18 days)	4	1	5
Opioids found in serum	Fentanyl and mitragynine	None	Fentanyl, heroin, and tramadol	Fentanyl, n-pyrrolidino- etonitazene, and tramadol	Fentanyl
**Other drugs detected in serum**					
Benzodiazepines	None	None	Bromazolam and clonazepam	Bromazolam, clonazolam, and etizolam	Bromazolam and etizolam
Stimulants	None	Methamphetamine	Cocaine	Methamphetamine	None
Other drugs	THC^§^	Olanzapine	Lidocaine, quinine, and xylazine	Lidocaine, quinine, and xylazine	Quinine and xylazine

**Abbreviations:** AMA = against medical advice; CPR = cardiopulmonary resuscitation; ED = emergency department; ICU = intensive care unit; THC = delta-9 tetrahydrocannabinol.

* Includes infusion dose given as the final dose.

^†^ Onset of withdrawal symptoms immediately after administration of medications used to treat opioid use disorder or overdose rather than from abstinence from opioids.

^§^ THC is the psychoactive constituent of cannabis.

## Preliminary Conclusions and Actions

These clinical findings are the first to illustrate the effects of medetomidine among a series of patients who used illicit drugs. Codetection with fentanyl is consistent with medetomidine use as an adulterant. Although knowledge about medetomidine’s short- and long-term effects is limited, permanent sequelae were not reported in any of the patients in this analysis. Xylazine was detected in samples from three patients, suggesting that medetomidine and xylazine exposure might occur from a concomitant drug exposure. Information regarding wounds was not recorded in the hospital chart in this cohort.

Hospitals are improving their treatment of patients with substance use disorders (*5*); however, continued support is required, given the low prevalence of naloxone distribution and addiction medicine referrals identified in this sample. Medetomidine is an emerging adulterant, associated in this report with fentanyl and other novel psychoactive substances, that is not detected as part of a standard urine drug screen. Although long-term, permanent sequelae were not reported, further investigation into the clinical and long-term effects of medetomidine is warranted.

## Footnotes

* https://www.cdc.gov/overdose-prevention/about/what-you-should-know-about-xylazine.html#cdc_generic_section_6-additional-resources

† Examples include abscess incisions, suture placement, and fracture reduction; or sedation of intubated patients.

§
https://www.cfsre.org/nps-discovery/public-alerts/medetomidine-rapidly-proliferating-across-usa-implicated-in-recreational-opioid-drug-supply-causing-overdose-outbreaks

¶ Chart reviews were performed by medical toxicology physicians or trained research assistants at each institution, using the patient’s electronic medical record. Abstracted data included details surrounding the overdose, demographic information, and clinical signs and symptoms including vital signs, medications administered, testing obtained, and the patient’s disposition.

** All suspected opioid overdose cases from participating sites that met inclusion criteria were eligible for inclusion in the study. Inclusion criteria were met for any patient aged >18 years with a suspected opioid overdose who had a leftover blood specimen for analysis. Persons who were incarcerated or patients with trauma or burns were excluded. All uploaded information was deidentified; therefore, some patients (e.g., those with approximately one overdose) might have been enrolled more than once. However, blood samples were only drawn once during each hospitalization (at the beginning) and thus should all represent a unique overdose. All five cases described in this report were associated with unique patients.
